# O8-7 Do physical activity, motor skills, and attention capacities predict the academic achievement of primary school children from disadvantaged neighbourhoods? Elaboration of inference conditional trees

**DOI:** 10.1093/eurpub/ckac094.063

**Published:** 2022-08-29

**Authors:** Caroline Bernal, Nicolas Fabre, Lena Lhuisset, Julien Bois

**Affiliations:** STAPS, Université de Pau et des Pays de l'Adour (UPPA), Tarbes, France

**Keywords:** children, motor skills, attentionnal capacities

## Abstract

**Background:**

In the 6-10 year period, physical activity (PA) and motor skills have a positive impact on cognitive development, which in turn act on academic performance. In order to better understand these links, a study was conducted to explore these relationships for disadvantaged children between the ages of 6 and 10.

**Methods:**

Children from two primary schools located in a disadvantaged neighbourhood (Tarbes, France) participated in the study. Variables were measured at 5 measurement times over 3 academic school years (2016; 2017; 2018). PA was measured by accelerometry over the whole day and weekend (MVPAF, MVPAWF). Motor skills were also assessed with a shuttle run test (NAV), a standing broad jump test (SBJ) and a tapping test (TT). A cardiorespiratory Shuttle Run test 20m (AR) was also carried out. Attentional capacities were measured with a computer-based Flanker Task: the Total Reaction Rime (RTT) of the correct answers (ms) for each child was collected. Finally, the children's score academic performance in French Language and Mathematics were collected. To study the relationships between the different variables, conditional inference trees including all these variables were performed with R software. Two trees were generated having as target variables respectively French language (FR) and Mathematics (MAT).

**Results:**

French language (FR) was predicted first by the age of the children (p > 0.001), but as well for the youngest and the oldest chidldren, FR was predicted by performance on the TT (p > 0.001) and RTT (p > 0.001): the children who perform better on the TT test and have a lower RTT are those who obtain the best results in French language. Mathematics (MAT) was explained by these same variables as for FR (p > 0.001). The evaluation of these two conditional inference tree by the Pseudo R-square were respectively 0.13 and 0.11.

**Discussion:**

These two conditional inference trees revealed that French language and Mathematics were predicted by attentional capacities (RTT), by motor variables (TT, NAV and SBJ). Finally, these models obtained were non-linear, complex and highlight different profiles of children. Although these relationships are documented in the literature (Diamond, 2002), this study confirms this from a longitudinal perspective, which is rarely used.

